# Association of Dietary Vitamin C and E Intake and Antioxidant Enzymes in Type 2 Diabetes Mellitus Patients

**DOI:** 10.5539/gjhs.v5n3p183

**Published:** 2013-03-20

**Authors:** Zahra Rafighi, Atena Shiva, Shahin Arab, Rokia Mohd Yusuf

**Affiliations:** 1 Department of Diabetic, Kamkar Hospital, Qom, Iran; 2 Department of Pathology, Oral and Maxillofacial Pathology Unit, Faculty of Dentistry, Mazandaran University of Medical Sciences, Sari, Iran; 3 Department of Clinical Biochemistry, Faculty of Medicine, Mazandaran University of Medical Sciences, Sari, Iran; 4 Departent of Nutrition and Dietetics, Faculty of Medicine and Health Sciences, University Putra Malaysia, Serdang, Malaysia

**Keywords:** T2DM, vitamin C supplementation, vitamin E supplementation, antioxidants

## Abstract

**Background:**

Diabetes mellitus consist of a various metabolic diseases such as hyperglycemia, increase glycosylated hemoglobin (HbA1c) and disorder in antioxidant enzymes activity, hence supplementing with antioxidant nutrients, mainly vitamin C and E seems to reduce oxidative injure in patients with type 2 diabetes mellitus (T2DM).

**Aim:**

To evaluate outcome of vitamin C and E supplementation on type 2 DM patients.

**Setting and Design:**

The study was completed in 170 T2DM on consumption of vitamin C, E, combination of C & E and placebo.

**Materials and Methods:**

The cases groups of this study consist of two major groups, which were named supplementation and placebo group. The group of supplementation consisted of 3 sub-groups, which received three capsules per day for a phase of three months. The parameters such as HbA1c, glucose, superoxide dismutase (SOD) and glutathione peroxides (GSH) were evaluated in baseline and after three months with supplementation.

**Statistical Analyses:**

The statistical analyses were evaluated with the use of mean ± SD, ANOVA-test and paired-sample t-test.

**Results:**

Mean age of 170 patients, 84 male and 86 female were 53.82±5.26 in the range of 30-60 years. The blood pressure results showed significant differences between the all supplement groups in baseline as compared to after receiving supplements (p<0.05). Use of vitamin C, E, and E & C showed significant differences in concentration of plasma FBS and HbA1c (p<0.05 & <0.001), but there was no significant differences in placebo groups. SOD and GSH enzymes levels showed a significant increased after consumption of vitamins in supplementation groups (p<0.001).

**Conclusion:**

This research confirmed that subjects with T2DM after three months supplementation of vitamins demonstrated significantly low level of hypertension, decrease levels of blood glucose, and increase SOD and GSH enzyme activity that can probably reduce insulin resistance by enhanced lowering oxidative stress parameters.

## 1. Introduction

Diabetes mellitus consist of a various metabolic diseases such as hyperglycemia, disturbances in glucose levels, and metabolism of lipid and protein that is resulted from disorder in secretion and/or act of insulin (Nathan, 2009), which has become a serious and common disease leading to many complications and premature death. Oxidative stress plays a central role in the onset of diabetes mellitus as well as in the development of vascular and neurological complications of the disease (AI-Nimer et al., 2012). In diabetes patients, reduction of activity antioxidant enzymes may show the identified sensitivity of these enzymes (GSH, SOD) to radical induced inactive. Consumption of antioxidants is connected with decreased risk of disease with T2DM (Montonen et al., 2004). Consumption of antioxidants nutrients, chiefly vitamin C and E seems to decrease oxidative injure associated with hyperglycemia and pancreatic β cell function and reduces the prevalence of diabetic complications (Wild et al., 2008). The most important benefit claimed for vitamins C, E, and their role as antioxidants, are scavengers of particles known as reactive oxygen species (ROS) (also sometimes called oxidants) (Gade et al., 2001). ROS are involved both in insulin signal transduction and in insulin resistance when produced in excess. Overfeeding, saturated fatty acids, and obesity play a key role in the excessive production of ROS. However, a diet rich in fruits and vegetables, and therefore antioxidants, has confirmed beneficial effects against oxidative damages and insulin resistance (Bishal et al., 2012). Reliable with this, vitamins supplementation was related with improved hyperlipidemia and decreased blood pressure (Caballero, 2004). Several researches showed that low levels of basal vitamin C level in diabetic patients can be resulted in increased oxidative stress parameters in T2DM patients (ADS, 2009). The structural of vitamin C is like to glucose, can change it in many chemical reactions, and therefore is useful in avoidance of non-enzymatic glycosylation reaction of proteins. The rate of T2DM has increased in the Islamic Republic of Iran therefore it is recommended that Iranian health policy-makers initiate more health promotional programmers and effective interventions. The current study was conducted to evaluate the property of antioxidant vitamins C and E supplements on biomarkers for risk of diabetes among T2DM patients.

## 2. Materials and Methods

### 2.1 Subjects

170 persons with T2DM were selected from Research Institute of Endocrinology and Metabolism, Iran University of Medical Sciences (IUMS) in this study. Patients with T2DM were chosen base on level of glucose more than 126 mg/dL (7.0 mmol/L), HbA1C equal or more than 6.5 mg/dL. It was necessary the patients were only treatment by oral medication, no insulin therapy and using vitamin and mineral supplements. The subjects with inflammatory diseases, lasting diseases occlusive, cardiovascular disease, chronic renal failure, range of age 30>age>65 years old and rang of BMI <25 (kg/m^2^), and Type 1 diabetes patients were excluded from the study. Statistical calculation of sample size is based on estimated prevalence of diabetes in Iran. The patients of this study were categorized with two major groups, supplementation and placebo group; which it were consisted of 3 sub-groups. In this regard, each supplementation group received three capsules per day for three months. The dosage consumption of vitamin C was (266.7 mg), vitamin E (300 IU), vitamin C+E (300 IU+266.7 mg) and placebo (placebo has been made of starch). Randomized assignment into the four groups was by proportional randomization method using a table of random numbers generated by Microsoft Excel. For example, if the sequence of the list generated by the computer is ABEDCCBBEDEA, then the first subject was randomized to the supplementation with vitamin C group, vitamin E group, vitamin C + E group, and placebo group until all the subjects were assigned to groups. After three months of supplementation with vitamin C, E and C & E, patients were examined again and the tests repeated. The supplement and placebo capsules looked the same and were particularly arranged for this study by Darou-Pakhsh (Tehran, Iran). After three months of supplementation with vitamins, patients were evaluated once more and the laboratory tests repeated. The research was agreed by the Ethic Committee of Faculty of Medicine and Health Science, University Putra Malaysia (UPM), and Iran University of Medical Sciences (IUMS). The knowledgeable permission was received from the patients at the first step before cooperating in this study. Source of Support was Mt grant 258.

### 2.2 Laboratory Measurements

About 20 ml blood samples were obtained under fasting situation into tubes with EDTA from patients with T2DM. The plasma were separated, aliquoted and then stored at -80°C until the analysis were done. For determination of antioxidant enzymes, 0.5 ml of patient’s whole blood were centrifuged for 10 minutes at 3000 rpm and then the plasma was aspirated and the erythrocytes were washed four times with 3 ml of 0.9% NaCl solution, centrifuged for 10 minutes at 3000 rpm after each washing. The washed red blood cells were then hemolyzed by freezing and thawing. Erythrocyte pellets can either be used fresh for analysis or stored at -80°C as required.

### 2.3 Routine Parameters

HbA1C parameter was analyzed by HPLC method (Sigma, USA). In determination of HbA1C, plasma extracts were combined and evaporated to dry under a stream of nitrogen, and the dry residue was dissolved in 0.25 ml of mobile phase and injected into the HPLC system. The separated hemoglobin components pass through the LED photometer flow cell where changes in absorbance are measured at 415 nm. The levels of vitamin C in the plasma were measured by spectrophotometer method using phenyl hydrazine indicator (Sigma, USA) as mentioned by (Ahmed et al., 2009). The vitamin E levels in plasma were measured using HPLC (HPLC, UK). Evaluation of superoxide dismutase (SOD) (U/ml) activity was examined by using a kit (Ransod kit Randox, USA). Glutathione peroxidase (GSH) was measured using a kit (Ransel; Randox USA). In the existence of glutathione reductase (GRX) and NADPH, the oxidized glutathione (GSSG) is directly converted to the reduced form with related oxidation of NADPH to NADP^+^ the reduce absorbance was determined at 340 nm.

### 2.4 Statistical Analyses

The data were calculated using the SPSS for window, version 16.0 (SPSS Inc., Chicago, IL, USA) and were explained as mean ± SD. Vitamin C, E and C & E intake were reported as the percentages, mean and standard deviation. Significance differences by treatment in antioxidant vitamins baseline and after three months within groups were determined by paired-sample t-test and between four groups by one-way analysis variance (ANOVA).

## 3. Results

Mean age of 170 patients with T2DM (84 male, 86 female) was 53.82±5.26 years (range: 30-60 yr). Of these, 44 patients were undergo supplementation with vitamin C, 43 patients vitamin E and 43 patients with vitamin C and E, and 40 patients received placebo. The result did not show significant differences in body mass index (BMI), waist circumference (WC), hip circumference (HC), and waist hip ratio (WHR) between groups, before and after supplementation. But the result in blood pressure parameters showed significant differences between the all supplement groups before as compared with after treatment (p<0.05). The subjects after receiving vitamin C, E, and E&C showed significant differences in plasma levels of FBS and HbA1c (P<0.05 & <0.001), but placebo groups did not show any changes in these parameters. The levels of SOD enzyme showed a significant improved after consumption of vitamin C, E and C & E in supplementation groups (p<0.001). The same results were received for GSH parameters which showed significant differences (p<0.001). In the current research, the levels of vitamin C and E parameters were evaluated that showed a significant increase after consumption (p<0.001).

## 4. Discussion

The data’s of the current study showed that T2DM patients showed significantly higher levels of SOD and GSH enzymes after treatment as compared to placebo groups that are in conformity with other studies (Adachi et al., 2004; Afkhami-Ardakani et al., 2009). The result of present study shows that taking three months supplementation vitamin of C, E and C+E caused significant decreased in fasting blood glucose at baseline and after three months. Previous study also showed that useful effects of oral vitamin C (1,000 mg/day for 4 month) can decrease FBS in T2DM patients (Afkhami-Ardakani, 2009; Chen, 2006). In agreement with Afkhami *et al* (2009), we found that high doses of oral vitamin C and E supplementation would improve level of glucose in T2DM patients (Dean, 2006). HbA1C showed significantly decreased levels in all receiving supplementation groups as compared to placebo after three months. On the contrary, Bishop and Schorah (2005) reported consumption of 500 mg vitamin C as well as placebos to 50 diabetic patients for 4 months, but found no significant difference in levels of HbA1C between groups, which can be mentioned, may be the low dose of vitamin C were used. In general, this study reveals that vitamin C and E can reduce the glycosylation of hemoglobin in patients with T2DM. Antioxidants have high superoxide scavenging activity, which with consumption of daily antioxidants, preferably vitamin C, E, and C+ E, can protect the body from oxidative damage (Brigelius, 2007). Furthermore, the mechanisms involved on antioxidants activity of vitamins C and E, they effect on free radicals and decrease oxidative damage, and also have potential role in raising antioxidant-related defenses in patients with diabetes (Bgelakovic, 2007). The low SOD activity in the diabetic groups could be the result of direct inhibition by H202 or could also is due to glycation of the enzyme (Adavhi et al., 2004). The results in this study revealed that SOD and GSH levels increased significantly in supplementation groups as compared with placebo group after supplementation of vitamins. The activity of SOD enzyme in the body is considered as one of the major enzymatic antioxidant defenses against superoxide radicals. Increases in SOD enzyme activity relates with improved resistance to oxidative stress (Herbeth et al., 2003). Soliman confirmed that standard dietary treatment in T2DM formed a considerable enhancement status of erythrocyte antioxidant and decreased serum and erythrocyte lipid peroxidation (Adachi et al., 2004; Soliman, 2008). On the other hand, GSH activities in supplementation groups with antioxidants were higher than the diabetic placebo groups, these changes may be in response to neutralize superoxide anions and offset oxidative stress. It is known that GSH reduces H202 in T2DM (Bhatia et al., 2003). The levels of plasma vitamin E reveal the amount of α-tocopherol in the body, which low plasma vitamin E levels were before observed in Type 2 diabetic patients Manzella et al., 2007). Vitamin E alone proved to be beneficial in decreasing the levels of free radicals and oxidative stress, it improve the action of insulin in patients with insulin resistance (Upritchard et al., 2008). In the other study recommended that consumption of vitamin E was associated with decreased HbA1c (Gowri et al., 2009). The combination of vitamin C and E were able to improve the action of endothelial only in T2DM. When vitamin E disarms a free radical, it becomes a weak free radical itself. But unlike bad free radicals, the vitamin E can be recycled, or turned back into an antioxidant, by vitamin C (Title et al., 2004; Rafighi et al., 2011).

## 5. Conclusion

The current study showed that the patients with T2DM after three months use of vitamins C, E and also combination of vitamins C and E showed significantly low level of hypertension, and improved insulin action and high level of SOD and GSH enzyme activity. Antioxidants have already shown to be potential role in the treatment of T2DM. Although, it seemed high level of oxidative stress parameter which was accompanied with low level of antioxidant enzymes in diabetes patients, these results may be very important with respect to the high morbidity and mortality rates in these patients. It may be possible that treatment by vitamin C, E and C & E as antioxidants can possibly reduce insulin resistance by improved condition of lowering oxidative stress parameters. On the other hand, suitable diet and treatment schedule may help in decreasing plasma glucose and increasing antioxidants capacity in type 2diabetes patients.
